# A Study of Individualized Diagnosis and Treatment for Depression with Atypical Features (iDoT-AFD): study protocol for a randomized clinical trial and prognosis study

**DOI:** 10.1186/s13063-023-07317-w

**Published:** 2023-05-04

**Authors:** Rubai Zhou, Huifeng Zhang, Shen He, Yi Li, Guiyun Xu, Jinsong Huang, Huaning Wang, Qian Wang, Biao Li, Xuemei Wang, Ningning Chen, Fang Li, Xiaosa Li, Mengjun Liu, Daihui Peng

**Affiliations:** 1 Division of Mood Disorders, Shanghai Mental Health Center, Shanghai Jiao Tong University School of Medicine, 600 South Wan Ping Road, Shanghai, 200030 China; 2 Wuhan Mental Health Center, Wuhan, Hubei China; 3 Department of Affective Disorders, Guangzhou Brain Hospital, Affiliated Hospital of Guangzhou Medical University, Guangzhou, China; 4 Dalian Seventh People’s Hospital, Dalian, Liaoning, China; 5 Department of Psychiatry, Xijing Hospital, Fourth Military Medical University, Xi’an, Shaanxi, China; 6 School of Biomedical Engineering, ShanghaiTech University, Shanghai, China; 7 Jianghan University, Wuhan, Hubei China; 8 School of Biomedical Engineering, Med-X Research Institute, Shanghai Jiao Tong University, Shanghai, China

**Keywords:** Major depressive disorder, Atypical features, Individualized diagnosis and treatment, Predictive biomarkers of conversion to bipolar disorder

## Abstract

**Background:**

Major depressive disorder (MDD) with atypical features, namely depression with atypical features (AFD), is one of the most common clinical specifiers of MDD, closely associated with bipolar disorder (BD). However, there is still a lack of clinical guidelines for the diagnosis, treatment, and prognosis of AFD. Our study mainly focuses on three issues about how to identify AFD, what is the appropriate individualized treatment for AFD, and what are the predictive biomarkers of conversion to BD.

**Methods:**

The Study of Individualized Diagnosis and Treatment for Depression with Atypical Features (iDoT-AFD) is a multicenter, prospective, open-label study consisting of a 12-week randomized controlled trial (RCT) and a continued follow-up until 4 years or reaching the study endpoint. It is enrolling 480 patients with AFD (120 per treatment arm), 100 patients with BD, and 100 healthy controls (HC). Multivariate dimension information is collected including clinical features, cognitive function, kynurenine pathway metabolomics, and multimodal magnetic resonance imaging (MRI) data. Firstly, multivariate informatics analyses are performed to recognize patients with AFD from participants including the first-episode and recurrent atypical depression, patients with BD, and patients with HC. Secondly, patients with atypical depression are randomly allocated to one of the four treatment groups including “single application of selective serotonin reuptake inhibitor (SSRI) or serotonin-noradrenaline reuptake inhibitor (SNRI)”, “SSRI/SNRI combined with mood stabilizer,” “SSRI/SNRI combined with quetiapine (≥ 150 mg/day),” or “treatment as usual (TAU)” and then followed up 12 weeks to find out the optimized treatment strategies. Thirdly, patients with atypical depression are followed up until 4 years or switching to BD, to explore the risk factors of conversion from atypical depression to BD and eventually build the risk warning model of conversion to BD.

**Discussion:**

The first enrolment was in August 2019. The iDoT-AFD study explores the clinical and biological markers for the diagnosis, treatment, and prognosis of AFD and further provides evidence for clinical guidelines of AFD.

**Trial registration:**

ClinicalTrials.gov NCT04209166. Registered on December 19, 2019.

**Supplementary Information:**

The online version contains supplementary material available at 10.1186/s13063-023-07317-w.

## Administrative information

Note: The numbers in curly brackets in this protocol refer to the SPIRIT checklist item numbers. The order of the items has been modified to group similar items (see http://www.equator-network.org/reporting-guidelines/spirit-2013-statement-defining-standard-protocol-items-for-clinical-trials/).

**Table:** 

Title {1}	A Study of Individualized Diagnosis and Treatment for Depression with Atypical Features (iDoT-AFD): study protocol for a randomized clinical trial and prognosis study
Trial registration {2a and 2b}	Clinical Trials.gov ID: NCT04209166, https://clinicaltrials.gov/ct2/show/NCT04209166. Registered on December 19, 2019.
Protocol version {3}	This paper is based on the study protocol version 03 of October 19, 2019.
Funding {4}	This trial is supported by the Key Clinical Research Program of Shanghai Mental Health Center (CRC2018ZD05) in relation to the design of the study, data collection, data analysis, and manuscript writing. Funding is also provided by Cross-Disciplinary and Translational Medical Research of Shanghai Jiao Tong University (ZH2018ZDA29) in relation to data collection and data analysis.
Author details {5a}	Rubai Zhou^1^, Huifeng Zhang^1^, Shen He^1^, Yi Li^2^, Guiyun Xu^3^, Jinsong Huang^4^, Huaning Wang^5^, Qian Wang^6^, Biao Li^2^, Xuemei Wang^7^, Ningning Chen^3^, Fang Li^4^, Xiaosa Li^5^, Mengjun Liu^8^, Daihui Peng^1*^ 1 Division of Mood Disorders, Shanghai Mental Health Center, Shanghai Jiao Tong University School of Medicine, Shanghai, China 2 Wuhan Mental Health Center, Wuhan, Hubei, China 3 Department of Affective Disorders, Guangzhou Brain Hospital, Affiliated Hospital of Guangzhou Medical University, Guangzhou, China 4 Dalian Seventh People’s Hospital, Dalian, Liaoning, China 5 Department of Psychiatry, Xijing Hospital, Fourth Military Medical University, Xi’an, Shaanxi, China 6 School of Biomedical Engineering, ShanghaiTech University, Shanghai, China 7 Jianghan University, Wuhan, Hubei, China 8 School of Biomedical Engineering, Med-X Research Institute, Shanghai Jiao Tong University, Shanghai, China
Name and contact information for the trial sponsor {5b}	This clinical trial is sponsored by Shanghai Mental Health Center, 600 South Wan Ping Road, Shanghai, 200030, China
Role of sponsor {5c}	The sponsor played no part in the study design; collection, management, analysis, and interpretation of the data; writing of the report; and decision to submit the report for publication.

## Introduction

### Background and rationale {6a}

Currently, there are about 340 million people with major depressive disorder (MDD) worldwide, and the lifetime prevalence rate of MDD is 3.9% in China [1]. The World Health Organization has reported that MDD would become the most serious global burden of disease and eventually turn into a public health problem in 2030 [2].

Depression is clinically heterogeneous, and based on the criteria of the Diagnostic and Statistical Manual of Mental Disorders, Fifth Edition (DSM-5), depressive disorders could be specified with eight kinds of clinical features. Atypical features were incorporated into the DSM-5 as an illness specifier, including mood reactivity, weight gain or appetite increase, hypersomnia, leaden paralysis, and interpersonal rejection sensitivity. MDD with atypical features, namely depression with atypical features (AFD), is one of the most common clinical specifiers of MDD accounting for 15~36% [3]. Rather than a classification of diseases, AFD is a distinctive group of MDD, regarded as a potential indicator for predicting bipolar disorder (BD) or even a variant of BD-II [4–6]. Through our BIPAS survey and review of the literature [7, 8], we found that there is still a lack of evidence from high-quality studies to enhance the clinical guidelines for the diagnosis, treatment, and prognosis of AFD.

From our previous studies, patients with MDD have dysfunctional neural circuits of the frontal cortex, amygdala, hippocampus, and cingulate gyrus, which are closely associated with clinical characteristics [9, 10]. Furthermore, it has been revealed that patients with AFD have increased brain perfusion in the frontal, temporal, and parietal cortex and decreased occipital perfusion [3]. Mediated by inflammatory factors, the tryptophan-kynurenine pathway (KP) can be catalyzed to trigger a series of cascade reactions, and the metabolite products will affect the content and function of neurotransmitters in the brain and participate in the development of depression [11, 12]. In our previous study, the metabolites of KP reduced significantly in patients with MDD and could be used as biomarkers for diagnosis with an accuracy of 83.6% [13]. Recent studies have shown that atypical symptoms are associated with higher levels of kynurenine and tryptophan [14]. Patients with AFD have been found to perform significantly differently than those with melancholic depression on cognitive tasks involving executive function, attention span and persistence, verbal and visual memory, and processing speed [15, 16]. Nevertheless, the findings of studies on AFD are still limited and controversial, possibly due to inconsistent inclusion criteria, small sample, and lack of follow-up. Up to now, no comprehensive clinical guidelines for AFD have been published. Accordingly, there is a need to probe into biomarkers for the diagnosis, treatment, and prognosis of AFD to provide evidence-based guidelines for patients with AFD.

The Study of Individualized Diagnosis and Treatment for Depression with Atypical Features (iDoT-AFD) is a multicenter, prospective, open-label study consisting of a 12-week randomized controlled trial (RCT) and a continued follow-up until 4 years or AFD switching to BD, which is designed based on our previous findings and recent advances in MDD. This paper describes the study protocol of the iDoT-AFD study. The protocol is based on the SPIRIT guidelines for clinical trials (Additional file 1) [17].

### Objectives {7}

The primary aim of the iDoT-AFD study is to identify the molecular imaging biomarkers for AFD by analysis of KP metabonomics and multimodal magnetic resonance imaging (MRI). The secondary aim of the iDoT-AFD study is to find out the optimized intervention and the predictive biomarkers of treatment effectiveness in patients with AFD. The third aim of the iDoT-AFD study is to screen out the risk factors and build a clinical early warning model to predict the conversion of AFD to BD.

### Trial design {8}

**Fig. 1:**
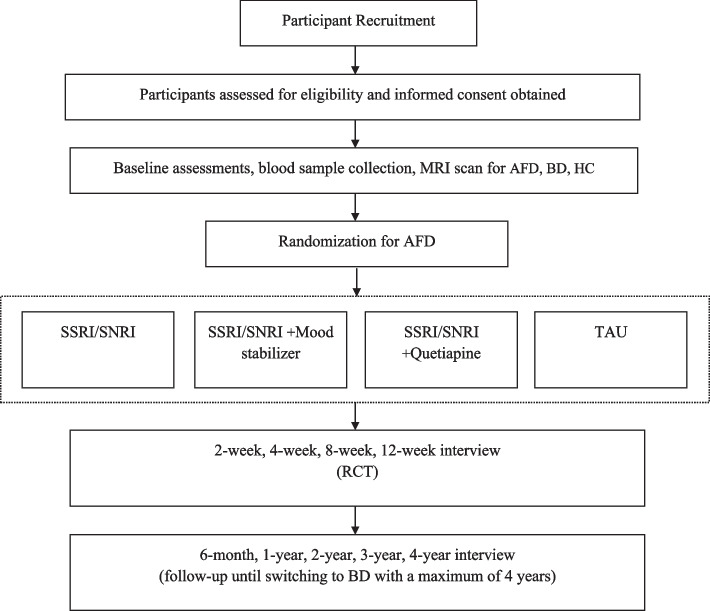
CONSORT diagram of the study AFD, depression with atypical features; BD, bipolar disorder; HC, healthy control; MRI, magnetic resonance imaging; SSRI, selective serotonin reuptake inhibitor; SNRI, serotonin-noradrenaline reuptake inhibitor; TAU, treatment as usual; RCT, randomized controlled trial

The iDoT-AFD study is a multicenter, exploratory, prospective, open-label study consisting of a 12-week parallel-group RCT with an allocation ratio of 1:1:1:1 to evaluate the clinical efficacy of four interventions and a prognosis study focusing on conversion to BD with a maximum of 4 years follow-up (Fig. 1).

## Methods: participants, interventions, and outcomes

### Study setting {9}

This clinical trial is sponsored by Shanghai Mental Health Centre (SMHC), 600 South Wan Ping Road, Shanghai, 200030, China. Five clinical sites are involved to recruit participants (SMHC, Wuhan Mental Health Center, the Affiliated Brain Hospital of Guangzhou Medical University, Dalian Seventh People’s Hospital, and Xijing Hospital of the Fourth Military Medical University), each with a principal investigator (PI) and a clinical trial coordinator (CTC). The iDoT-AFD study also includes biomedical engineering experts from Shanghai Jiao Tong University and ShanghaiTech University to ensure high-level data mining.

### Eligibility criteria {10}

The inclusion criteria for patients with AFD are as follows: (1) 16–60 years old, (2) meeting the DSM-5 criteria for MDD, (3) scored 20 or higher on the Hamilton’s Depression Scale with 24 items (HAMD-24), (4) enough audiovisual ability and comprehensive ability to accomplish the interview, (5) necessary and appropriate to accept the treatment of antidepressants, (6) scored less than 14 on the Hypomania Symptom Checklist-32 (HCL-32), and (7) meeting DSM-5 specifier for atypical features: mood reactivity and 2 or more of the following—significant weight gain or increase in appetite, hypersomnia, leaden paralysis, and a long-standing pattern of interpersonal rejection sensitivity that results in significant social or occupational impairment.

The exclusion criteria for patients with AFD are as follows: (1) severe medical or neurological problems; (2) previous episodes of mania or hypomania; (3) female patients who are pregnant, planning to be pregnant, or breastfeeding; (4) high risk of suicide; (5) had ECT, MECT, or rTMS in the past 6 months; and (6) experienced severe personality disorder, mental retardation, and anorexia/bulimia nervosa.

Additionally, all included patients with AFD should be drug-naive or medication free for more than 1 week.

The inclusion criteria for patients with BD are as follows: (1) 16–60 years old, (2) meeting the DSM-5 criteria for major depressive episode of bipolar I disorder or bipolar II disorder, (3) scored 20 or higher on the HAMD-24, and (4) enough audiovisual ability and comprehensive ability to accomplish the interview.

The exclusion criteria for patients with BD are as follows: (1) severe medical or neurological problems; (2) female patients who are pregnant, planning to be pregnant, or breastfeeding; (3) high risk of suicide; (4) had electroconvulsive therapy, modified electroconvulsive therapy, or repetitive transcranial magnetic stimulation in the past 6 months; and (5) experienced severe personality disorder, mental retardation, and anorexia/bulimia nervosa.

The inclusion and exclusion criteria for HC are as follows: (1) 16–60 years old, (2) enough audiovisual ability and comprehensive ability to accomplish the interview, and (3) no history of mental disorder or serious somatic disease.

### Who will take informed consent? {26a}

The principal investigators (PIs), or trained investigators, who are fellows or residents in the clinical sites and delegated by the PIs, introduce the information about the iDoT-AFD study to potential participants. If any participant is willing to participate in the clinical trial, the informed consent will be signed before the eligibility screening.

### Additional consent provisions for collection and use of participant data and biological specimens {26b}

Blood sample collection and MRI scan have been clarified in the informed consent so no additional consents are provided.

### Interventions

#### Explanation for the choice of comparators {6b}

Treatment as usual (TAU) is used as a comparator. TAU allows any pharmacotherapy concordant with major practice guidelines for MDD.

#### Intervention description {11a}

All eligible patients with AFD are randomly allocated to one of the four treatment groups including “single application of selective serotonin reuptake inhibitor (SSRI) or serotonin-noradrenaline reuptake inhibitor (SNRI),” “SSRI/SNRI combined with mood stabilizer,” “SSRI/SNRI combined with quetiapine (≥ 150 mg/day),” or “TAU.” Mood stabilizer only means classical drugs including lithium and anticonvulsants. Quetiapine is chosen from atypical antipsychotics as suggested by guidelines [18].

#### Criteria for discontinuing or modifying allocated interventions {11b}

When recruited, participants are informed that they can reject to participate or leave the study at any time including but not limited to the following reasons.

1. The participants withdraw consent to continue the study.
2. The participants fail to accomplish the interview or have an unplanned pregnancy.
3. It is inappropriate to continue the therapeutic schedule because of poor efficacy or intolerant side effects.
4. Participants develop any serious adverse events possibly correlated with the interventions or anything else of the study.
5. Participants develop serious suicidal ideation or risk of suicidal behavior.

#### Strategies to improve adherence to interventions {11c}

Every participant has a fixed investigator for assessments at baseline and follow-up, and the investigator will keep in touch with the participant to provide encouragement and remind the patients of taking medicine.

#### Relevant concomitant care permitted or prohibited during the trial {11d}

Benzodiazepines and similar hypnotics are allowed for short-term use with careful records.

#### Provisions for post-trial care {30}

There is no anticipated harm and compensation for trial participation.

### Outcomes {12}

The following is the primary outcome:

1. Clinical remission (examiner-rating), defined as total scores less than or equal to 7 on the HAMD-24 at the end of the 12th week

The following are the secondary outcomes:

1. Clinical remission (self-rating), defined as total scores less than or equal to 5 on the QIDS-SR16 at the end of the 12th week
2. Early improvement, defined as a ≥ 20% decrease from the baseline HAMD-24 at the end of the 2nd week
3. Treatment response, defined as a ≥ 50% decrease from the baseline HAMD-24 at the end of the 12th week
4. Severity and incidence of adverse events
5. The time of treatment discontinuation for any cause
6. Working or learning time lost to any cause associated with the present study

### Participant timeline {13}

**Fig. 2:**
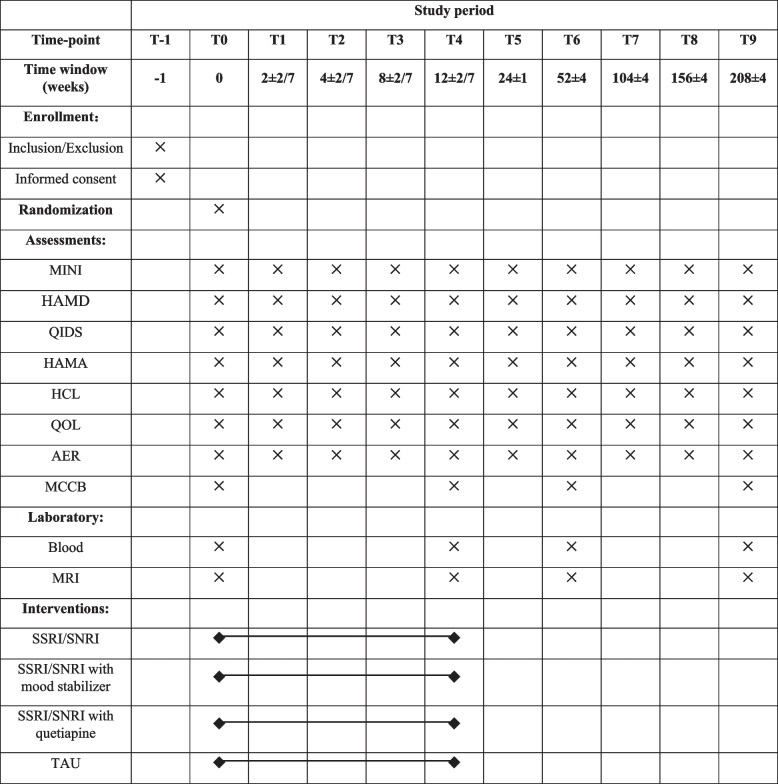
Participant timeline MCCB, MATRICS Consensus Cognitive Battery; MRI, magnetic resonance imaging; MINI, M.I.N.I International Neuropsychiatric Interview; HAMD, Hamilton’s Depression Scale with 24 items; QIDS, Quick Inventory of Depressive Symptomatology (self-report) with 16 items; HAMA, Hamilton’s Anxiety Scale; HCL, Hypomania Symptom Checklist-32; QOL, Quality of Life Scale with 6 items; AER, adverse events report; SSRI/SNRI, selective serotonin reuptake inhibitor/serotonin-noradrenaline reuptake inhibitor; TAU, treatment as usual

The participant timeline is shown in Fig. 2.

### Sample size {14}

The sample size estimation refers to the primary outcome. A clinically relevant medium effect size (*d* = 0.5) is expected to be found in a two-sample *t*-test of at least one of the three interventions compared to TAU when calculating the sample size. Using the power of 1−*β* = 80% and the significance level of *α* = 0.05 (two-sided), 63 participants are needed per group. Considering a drop-out rate of ≤ 30%, 90 participants need to be allocated to each group. Finally, we planned to recruit 120 patients per group for a total of 480 included patients with AFD.

### Recruitment {15}

The iDoT-AFD study enrolls 480 patients with AFD (120 per treatment group), 100 patients with BD, and 100 healthy participants.

### Assignment of interventions: allocation

#### Sequence generation {16a}

For each center, a randomization list has been made using software-generated randomized numbers by a research assistant without clinical participation in the trial. Randomization is stratified for each clinical site. A block randomization is conducted in an allocation ratio of 1:1:1:1 with a fixed block length per center.

#### Concealment mechanism {16b}

The randomization codes are packed into sealed envelopes in sequence. The envelopes are locked in a cabinet by a research nurse per center, and each envelope will be opened in ascending order to assign intervention after a patient with AFD is recruited.

#### Implementation {16c}

The research assistant without clinical participation in the trial will generate the allocation sequence. Trained investigators will enroll participants. The research nurse at each center will keep and open the envelopes containing randomization codes.

### Assignment of interventions: blinding

#### Who will be blinded {17a}

The iDoT-AFD is an open-label study without blinding.

#### Procedure for unblinding if needed {17b}

The iDoT-AFD is an open-label study without blinding so unblinding will not occur.

### Data collection and management

#### Plans for assessment and collection of outcomes {18a}

M.I.N.I International Neuropsychiatric Interview (MINI) [19] is used to conduct a comprehensive screening. HAMD-24 and Quick Inventory of Depressive Symptomatology (self-report) with 16 items (QIDS-SR16) [20] are applied to evaluate depressive symptoms, Hamilton’s Anxiety Scale (HAMA) [21] is used to assess anxious symptoms, HCL-32 is performed to recognize mania or hypomania symptoms, and Quality of Life Scale with 6 items (QOL-6) [22] is used to evaluate the life quality briefly. Additionally, family history, physical or mental comorbidity, the medication used before, prescription for this time, number of episodes, first-onset time and age, and duration of illness are also questioned and recorded. Adverse events are recorded in adverse events report (AER) forms when needed during the follow-up period. Cognitive function is assessed using the MATRICS Consensus Cognitive Battery (MCCB) [23]. MCCB consists of 10 subtests that mainly reflect the cognitive function of 7 cognitive domains including speed of processing, attention and vigilance, working memory, verbal learning, visual learning, reasoning and problem-solving, and social cognition.

After the 12-week randomized intervention, patients with AFD will be followed up until switching to BD for a maximum of 4 years. Face-to-face interviews, including clinical and neurocognitive assessments, MRI scans, and blood specimen collection, will be scheduled for 12 weeks, 1 year, and 4 years. The interim interviews of clinical assessments will be performed at 2 weeks, 4 weeks, 8 weeks, 6 months, 2 years, and 3 years through offline or online approaches. Patients with AFD will undergo a semi-structured interview at the follow-up time points to determine whether they have experienced mania or hypomania or have been diagnosed with bipolar disorder by psychiatrists during the interval since the previous follow-up. If a patient has experienced mania or hypomania or has been diagnosed with bipolar disorder by psychiatrists during the interval, multidimensional data will be recollected and follow-up will be terminated.

#### Plans to promote participant retention and complete follow-up {18b}

All participants will be generally informed about the significance of follow-up and the value of their contribution before being involved. The investigator will explain the results of the assessments and examinations at every follow-up time point, which may help the participants know more about their mental health condition.

#### Data management {19}

In the iDoT-AFD study, all data are collected electronically using the electronic case report form (eCRF) and synchronized in real-time among all the clinical sites. In this way, the data manager can conduct quality reviews of the database timely. A standardized operation manual of the iDoT-AFD study will be provided to all the investigators of the study. All investigators will be trained about the assessments and study procedures every 6 months to ensure consistent and reliable data collection. The leading PI will have access to the complete trial data and can decide the access to the database.

#### Confidentiality {27}

Participants’ personal information will be managed to maintain confidentiality. The participants’ identification code rather than their names will be used for recording and classifying the eCRF. Only the research associates and IRB have the access to the original medical records to verify the reliability of the data in the trial.

#### Plans for collection, laboratory evaluation, and storage of biological specimens for genetic or molecular analysis in this trial/future use {33}

##### Assessments of metabolic factors in the kynurenine pathway

Five-milliliter venous blood fasting for more than 4 h is collected from participants using the ethylene diamine tetraacetic acid (EDTA) anticoagulant tube. After placing at normal temperature for 30 min, the blood samples are centrifuged at the rate of 3000 rpm, and the plasma is separated and then stored at − 80 °C. All specimens will be examined and analyzed together after finishing collection using ultra-performance liquid chromatography-tandem mass spectrometry.

##### Neuroimaging assessments

Neuroimaging data are acquired using the 3.0-T scanner at available clinical sites. Imaging data including structural MRI, rest state functional MRI, and diffusion tensor imaging (DTI)/diffusion kurtosis imaging (DKI) are collected. All participants are planned to be scanned at baseline, and patients with AFD will also be scanned at 12 weeks, 1 year, and 4 years.

## Statistical methods

### Statistical methods for primary and secondary outcomes {20a}

To evaluate the effectiveness of the 12-week treatment for patients with AFD, we plan to perform an intention-to-treat analysis to pursue our primary outcome. A mixed-effect model for repeated measures will be applied to analyze the main effects of interventions, time, and the interaction on HAMD total scores to find the optimal treatment for patients with AFD. Covariates like gender, age, education, and the baseline severity of depressive symptoms will be added to the model to correct the potential confounding effects.

### Interim analyses {21b}

There was no design of any interim analyses because the safety and effectiveness of all four interventions have been proved for patients with MDD in previous studies.

### Methods for additional analyses (e.g., subgroup analyses) {20b}

To explore the potential biomarker identifying AFD, analysis of variance (ANOVA), Kruskal–Wallis tests, or chi-square tests will be applied to compare the group differences of multivariate-dimension information among the first-episode AFD patients, recurrent AFD patients, and patients with BD and HC.

The prediction model of patients with AFD switching from depression to mania or hypomania will be constructed. Multidimensional data obtained at baseline and their changes during the follow-up period will be used to predict the binary outcome (switching to mania/hypomania or not). The concordance index will be calculated to assess the discrimination of the model. Construction of the multivariate prediction model will be conducted by collaborators specializing in biomedical engineering.

### Methods in analysis to handle protocol non-adherence and any statistical methods to handle missing data {20c}

The missing data will be managed using multiple imputation techniques. Sensitivity analysis will be further conducted using data from complete cases and data with replacing values.

### Plans to give access to the full protocol, participant-level data, and statistical code {31c}

The datasets analyzed during the current study and statistical code are available from the corresponding author upon reasonable request, as is the full protocol.

### Oversight and monitoring

#### Composition of the coordinating center and trial steering committee {5d}

The coordinating center is in charge of monitoring, data management, and statistical analysis. SMHC is responsible for designing the trial and making the analysis plan.

#### Composition of the data monitoring committee, its role, and reporting structure {21a}

An independent data monitoring committee (DMC) comprises members specializing in psychiatry, statistics, and ethics. DMC monitors the recruitment and follow-up of participants, the adverse effect reports, the trial outcomes, the safety and efficacy of the interventions, the quality of the CRF data, and the general conduction of the iDoT-AFD study.

#### Adverse event reporting and harms {22}

The investigators will record all the adverse events and evaluate the severity.

#### Frequency and plans for auditing trial conduct {23}

Trial audits by the representatives from the quality assurance department of SMHC are conducted every half year or any time necessary following the regulatory guidance.

#### Plans for communicating important protocol amendments to relevant parties (e.g., trial participants, ethical committees) {25}

Any protocol modification will be sent to the principal investigator (PI) to add to the investigator files. Protocol amendments will also be reported to IRB and updated in the clinical trial registry.

#### Dissemination plans {31a}

The study findings will be disseminated through presentations at the national and international scientific conferences, publications in peer-reviewed journals in psychiatry or neuroscience, evidence‑based clinical guidelines or recommendations, and textbooks on the diagnosis, treatment, and prognosis of AFD. The study data will not be shared for any purpose without the permission of the IRB and PI.

## Discussion

AFD is one of the most common clinical specifiers of MDD, closely associated with BD. The iDoT-AFD study mainly aims to find out the molecular imaging biomarkers for identifying AFD, optimize the individualized treatment for patients with AFD, and build a clinical early warning model to predict the conversion of AFD to BD. Participants are recruited from 5 clinical sites in the eastern, western, southern, northern, and central regions of China to assure a broadly representative population. Accordingly, the findings of the iDoT-AFD study would be also widely characteristic and generalizable. The iDoT-AFD study is a multicenter, prospective, randomized study, which can provide evidence for clinical guidelines for the diagnosis, treatment, and prognosis of AFD.

### Limitation

Except for drug-naive patients, patients with AFD who are medication-free for more than 1 week are also recruited. A washout period of 1 week is relatively short and may influence the interpretation of intervention effects. Subgroup analyses will be performed to clarify the influence, and the washout period will be more carefully determined in future studies.

## Trial status

This paper is based on the study protocol version 03 of October 19, 2019. The iDoT-AFD study was registered in ClinicalTrials.gov using ID NCT04209166 on December 19, 2019. The first enrollment of participants was on August 13, 2019; the recruitment is planned to be completed before October 2022; and the follow-up will be continued until 4 years later or all participants reach the study endpoint.

## Supplementary Information

**Additional file 1.**  SPIRIT Checklist for iDoT-AFD study.

## Data Availability

The datasets analyzed during the current study and statistical code are available from the corresponding author upon reasonable request, as is the full protocol.

## Abbreviations

iDoT-AFD: Individualized Diagnosis and Treatment for Depression with Atypical Features
AFD: Depression with atypical features
MDD: Major depressive disorder
BD: Bipolar disorder
HC: Healthy control
RCT: Randomized controlled trial
DSM-5: Diagnostic and Statistical Manual of Mental Disorders, Fifth Edition
BIPAS: National Bipolar Mania Pathway Survey
KP: Kynurenine pathway
EDTA: Ethylene diamine tetraacetic acid
MRI: Magnetic resonance imaging
SMHC: Shanghai Mental Health Centre
PI: Principal investigator
CTC: Clinical trial coordinator
HAMD-24: Hamilton’s Depression Scale with 24 items
HCL-32: Hypomania Symptom Checklist-32
SSRI: Selective serotonin reuptake inhibitor
SNRI: Serotonin-noradrenaline reuptake inhibitor
TAU: Treatment as usual
MINI: M.I.N.I International Neuropsychiatric Interview
QIDS-SR16: Quick Inventory of Depressive Symptomatology (self-report) with 16 items
HAMA: Hamilton’s Anxiety Scale
QOL-6: Quality of Life Scale with 6 items
MCCB: MATRICS Consensus Cognitive Battery
DTI: Diffusion tensor imaging
DKI: Diffusion kurtosis imaging
ANOVA: Analysis of variance
DMC: Data monitoring committee
eCRF: Electronic case report form

## References

[1] Huang Y, Wang Y, Wang H, Liu Z, Yu X, Yan J (2019). Prevalence of mental disorders in China: a cross-sectional epidemiological study. Lancet Psychiatry.

[2] Whiteford HA, Degenhardt L, Rehm J, Baxter AJ, Ferrari AJ, Erskine HE (2013). Global burden of disease attributable to mental and substance use disorders: findings from the Global Burden of Disease Study 2010. Lancet (London, England).

[3] Lojko D, Rybakowski JK (2017). Atypical depression: current perspectives. Neuropsychiatr Dis Treatment.

[4] Peng D, Huang Y, Jiang K (2016). Atypical features and bipolar disorder. Shanghai Arch Psychiatry.

[5] Akiskal HS, Benazzi F (2005). Atypical depression: a variant of bipolar II or a bridge between unipolar and bipolar II?. J Affect Disord.

[6] Mitchell PB, Wilhelm K, Parker G, Austin MP, Rutgers P, Malhi GS (2001). The clinical features of bipolar depression: a comparison with matched major depressive disorder patients. J Clin Psychiatry..

[7] Peng D, Shen T, Byrne L, Zhang C, Huang Y, Yu X (2015). Atypical features and treatment choices in bipolar disorders: a result of the National Bipolar Mania Pathway Survey in China. Neurosci Bull.

[8] Chinese Academy of Depressive Disorders CSoP (2021). Recommendations of clinical evaluation, diagnosis, and treatment for patients of major depressive disorder with atypical features. Chin J Psychiatry.

[9] Peng D, Shi F, Shen T, Peng Z, Zhang C, Liu X (2014). Altered brain network modules induce helplessness in major depressive disorder. J Affect Disord.

[10] Iwabuchi SJ, Peng D, Fang Y, Jiang K, Liddle EB, Liddle PF (2014). Alterations in effective connectivity anchored on the insula in major depressive disorder. Euro Neuropsychopharmacol.

[11] Schmidt HD, Shelton RC, Duman RS (2011). Functional biomarkers of depression: diagnosis, treatment, and pathophysiology. Neuropsychopharmacology.

[12] Sublette ME, Galfalvy HC, Fuchs D, Lapidus M, Grunebaum MF, Oquendo MA (2011). Plasma kynurenine levels are elevated in suicide attempters with major depressive disorder. Brain Behav Immunity.

[13] Liu H, Ding L, Zhang H, Mellor D, Wu H, Zhao D (2018). The metabolic factor kynurenic acid of kynurenine pathway predicts major depressive disorder. Front Psychiatry.

[14] Milaneschi Y, Allers KA, Beekman ATF, Giltay EJ, Keller S, Schoevers RA (2021). The association between plasma tryptophan catabolites and depression: the role of symptom profiles and inflammation. Brain Behav Immunity.

[15] Bosaipo NB, Foss MP, Young AH, Juruena MF (2017). Neuropsychological changes in melancholic and atypical depression: a systematic review. Neurosci Biobehav Rev.

[16] Lin K, Xu G, Lu W, Ouyang H, Dang Y, Lorenzo-Seva U (2014). Neuropsychological performance in melancholic, atypical and undifferentiated major depression during depressed and remitted states: a prospective longitudinal study. J Affect Disord.

[17] Chan AW, Tetzlaff JM, Gøtzsche PC, Altman DG, Mann H, Berlin JA (2013). SPIRIT 2013 explanation and elaboration: guidance for protocols of clinical trials. BMJ (Clin Res Ed).

[18] Yatham LN, Kennedy SH, Parikh SV, Schaffer A, Bond DJ, Frey BN (2018). Canadian Network for Mood and Anxiety Treatments (CANMAT) and International Society for Bipolar Disorders (ISBD) 2018 guidelines for the management of patients with bipolar disorder. Bipolar Disord.

[19] Sheehan DV, Lecrubier Y, Sheehan KH, Amorim P, Janavs J, Weiller E (1998). The Mini-International Neuropsychiatric Interview (M.I.N.I.): the development and validation of a structured diagnostic psychiatric interview for DSM-IV and ICD-10. J Clin Psychiatry..

[20] Rush AJ, Trivedi MH, Ibrahim HM, Carmody TJ, Arnow B, Klein DN (2003). The 16-Item Quick Inventory of Depressive Symptomatology (QIDS), clinician rating (QIDS-C), and self-report (QIDS-SR): a psychometric evaluation in patients with chronic major depression. Biol Psychiatry.

[21] Hamilton M (1959). The assessment of anxiety states by rating. Br J Med Psychol.

[22] Phillips MR, Yang G, Zhang Y, Wang L, Ji H, Zhou M (2002). Risk factors for suicide in China: a national case-control psychological autopsy study. Lancet (London, England).

[23] Liang S, Yu W, Ma X, Luo S, Zhang J, Sun X (2020). Psychometric properties of the MATRICS Consensus Cognitive Battery (MCCB) in Chinese patients with major depressive disorder. J Affect Disord.

